# Rare Thyroid Cartilage and Diaphragm Metastases from Lung Cancer Visualized on F-18 FDG-PET/CT Imaging

**DOI:** 10.4274/MIRT.019882

**Published:** 2011-08-01

**Authors:** Pelin Özcan Kara, Gonca Kara Gedik, Oktay Sarı, Orhan Özbek

**Affiliations:** 1 Selçuk University, Selçuklu Medical Faculty, Department of Nuclear Medicine, Selçuklu, Konya, Turkey; 2 Selçuk University, Meram Medical Faculty, Department of Nuclear Medicine, Meram, Konya, Turkey; 3 Selçuk University, Meram Medical Faculty, Department of Radiology, Meram, Konya, Turkey

**Keywords:** PET/CT; Lung cancer; Diaphragm metastasis; Thyroid cartilage metastasis

## Abstract

Positron emission tomography (PET) with F-18 fluorodeoxyglucose (FDG) has evolved as a useful imaging modality in the assessment of a variety of cancers, especially for tumor staging and post treatment monitoring. It provides metabolic information. Although, when used alone, relative lack of anatomic landmarks, is a major limitation of PET imaging, this limitation of PET imaging is overcome by the availability of integrated PET/CT imaging. PET and CT images are acquired in one procedure, yielding fused anatomical and functional data sets. Studies with integrated PET/CT imaging have shown promising results. In this case, we present an interesting integrated PET/CT imaging in a lung cancer patient with rare, diaphragm and thyroid cartilage metastases.

**Conflict of interest:**None declared.

## INTRODUCTION

Positron emission tomography (PET) with F-18 fluorodeoxyglucose (FDG) has evolved as a useful imaging modality in the assessment of a variety of cancers, especially for tumor staging and post treatment monitoring. It provides metabolic information. Although, when used alone, relative lack of anatomic landmarks, is a major limitation of PET imaging, this limitation of PET imaging is overcome by the availability of integrated positron emission tomography/computed tomography (PET/CT) imaging. PET and CT images are acquired in one procedure, yielding fused anatomical and functional data sets. In this case, we present an interesting integrated PET/CT imaging in a lung cancer patient with rare, diaphragm and thyroid cartilage metastases.

## CASE REPORT

**Figure 1:**
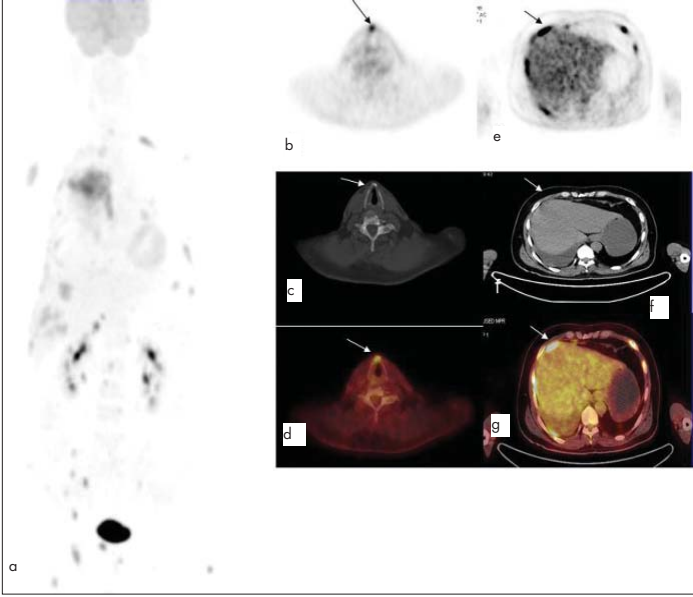
MIP image of PET/CT (a) demonstrated a right upper lobemass (SUVmax: 28.0), left lower lobe lung nodule (SUV_max_: 3.6),paracardiac lymph node (SUV_max_: 6.2), bilateral adrenal lesions(SUVmax: 18.3-right and 4.7-left), intraabdominal lymph nodes(SUV_max_: 5.6), a 12 mm lesion on anterior thyroid cartilage withdestruction on CT imaging (SUV_max_: 6.5) (a,b,c,d) and multiple bonelesions with increased FDG uptake were detected. Additionally,PET/CT showed areas of increased tracer uptake between rightlower mediastinum and liver. The ill-defined lesions adjacent to theliver were missed on CT. The exact localization of these lesions couldonly be determined on PET/CT imaging that could be diagnosed asmetastases to the diaphragm (a,e,f,g)

A 49 year-old man was referred to our institution for initial staging of non-small cell lung cancer (NSCLC). The patient underwent PET/CT imaging 60 minutes after 10 mCi FDG injection (Figure 1). MIP image of PET/CT (a) demonstrated a 85x111x88 mm right upper lobe mass with increased FDG uptake. Left lower lobe lung nodule with a diameter of 10 mm, paracardiac lymph node, right and left adrenal lesions, paraaortic and paraceliac lymph nodes, a 12 mm lesion on anterior thyroid cartilage with destruction on CT imaging (SUV_max_: 6.5) (a,b,c,d) and multiple bone lesions with increased FDG uptake were detected. Additionally, PET/CT showed areas of increased tracer uptake between right lower mediastinum and liver. The ill-defined lesions adjacent to the liver were missed on CT. The exact localization of these lesions could only be determined on PET/CT imaging that could be diagnosed as metastases to the diaphragm (a,e,f,g).

## LITERATURE REVIEW AND DISCUSSION

Neither diaphragm metastasis nor thyroid cartilage metastasis was proved histopathologically, because performing biopsy in a stage IV lung cancer patient with widespread metastasis to prove diaphragm or thyroid cartilage metastasis would be a quite invasive approach. In the absence of histopathological diagnosis, normal variations in the pattern of FDG uptake, as well as physiological variants and benign pathologic conditions of FDG accumulation should not be confused with metastatic disease. It is important to recognize the increased FDG uptake in diaphragm in patients with pulmonary pathology. Increased uptake in the diaphragm and especially at the crura is mostly thought to be secondary to hyperventilation. The high FDG uptake in these situations is typically seen bilaterally (1). Unilateral FDG uptake in the diaphragm and crura was also reported (2). However, in our patient there was no bilateral or unilateral FDG uptake in the crura of the diaphragm. The infradiaphragmatic foci of increased FDG activity in the upper abdomen, poses a diagnostic dilemma on PET imaging, especially when they are asymmetrical or focal or did not conform to the expected physiological tracer distribution (3). By localizing these sites to normal fatty tissues, fused PET/CT images exclude benign nature of this uptake. The uptake pattern in thyroid cartilage may have been developed because of asymmetrical vocal cord uptake or a glottic second primary of laryngeal cancer. But these were excluded in fused PET/CT images.

Histologically, lung cancer is divided into small cell lung carcinoma (15% to 20%) and non-small cell lung carcinoma. NSCLC comprises about 80% of all lung cancers. PET is a standard modality for both mediastinal and distant staging of NSCLC. The presence of distant metastases categorizes the patient as having stage IV disease. The most common distant metastases in NSCLC are the adrenal glands, skeleton, brain and liver (4). Unsuspected distant metastases will be detected by PET imaging in ˜ %10 of patients (5). In this case report, rare diaphragm and thyroid cartilage metastases of lung cancer were illustrated. Metastases of malignant tumors to cartilaginous tissue due to deprivation of vessels and to diaphragm are extremely rare. In a report by Wiesenthal AA et al, rare thyroid cartilage metastasis in a multipl myeloma patient with diffuse osseous and extramedullary lesions was shown by fused PET/CT imaging (6). In another case report by Lee KH et al, isolated diaphragmatic metastasis originated from adenocarcinoma of the colon was reported (7). Although our case was already at stage IV, PET/CT could very well demonstrated rare and unexpected metastatic cancer and provided more accurate staging in a lung cancer patient. PET/CT revealed more lesions in the patient than either PET or CT alone. Additionally, it permitted the exact anatomic localization of pathological tracer uptake.
